# In Vitro 3D Cultures to Model the Tumor Microenvironment

**DOI:** 10.3390/cancers13122970

**Published:** 2021-06-13

**Authors:** Fabrizio Fontana, Monica Marzagalli, Michele Sommariva, Nicoletta Gagliano, Patrizia Limonta

**Affiliations:** 1 Department of Pharmacological and Biomolecular Sciences, University of Milan, Via Balzaretti 9, 20133 Milan, Italy; monica.marzagalli@unimi.it (M.M.); patrizia.limonta@unimi.it (P.L.); 2 Department of Biomedical Sciences for Health, University of Milan, Via Mangiagalli 31, 20133 Milan, Italy; michele.sommariva@unimi.it (M.S.); nicoletta.gagliano@unimi.it (N.G.)

**Keywords:** cell culture, in vitro, 3D, tumor microenvironment, stroma, extracellular matrix, angiogenesis, fibroblasts, immune system, cancer therapy

## Abstract

**Simple Summary:**

Tumor stroma is known to significantly influence cancer initiation and progression. In the last decade, 3D cell cultures have shown potential in modeling the tumor microenvironment. This review summarizes the main features of current 3D models, shedding light on their importance in the study of cancer biology and treatment.

**Abstract:**

It is now well established that the tumor microenvironment plays a key role in determining cancer growth, metastasis and drug resistance. Thus, it is fundamental to understand how cancer cells interact and communicate with their stroma and how this crosstalk regulates disease initiation and progression. In this setting, 3D cell cultures have gained a lot of interest in the last two decades, due to their ability to better recapitulate the complexity of tumor microenvironment and therefore to bridge the gap between 2D monolayers and animal models. Herein, we present an overview of the 3D systems commonly used for studying tumor–stroma interactions, with a focus on recent advances in cancer modeling and drug discovery and testing.

## 1. Introduction

The tumor stroma is composed of extracellular matrix (ECM) and various non-malignant cell types, including endothelial, mesenchymal (e.g., fibroblasts and adipocytes) and immune (e.g., lymphocytes, monocytes and neutrophils) cells [1,2]. These cells not only communicate with each other but also with the tumor mass, by secreting a variety of molecules that affect tumor behavior through different signaling pathways [3,4]. Among them, several growth factors, such as epidermal growth factor (EGF), fibroblast growth factor (FGF), platelet-derived growth factor (PDGF), transforming growth factor (TGF) and vascular endothelial growth factor (VEGF), have been widely recognized as master regulators of the interactions occurring within the tumor microenvironment [5]. In addition to these polypeptides, cytokines, extracellular vesicles and miRNAs also play a pivotal role in the control of cell–cell communication [6,7,8]. In this context, emerging evidence suggests that the activated stroma represents a crucial modulator of cancer growth, migration, angiogenesis, immunosurveillance evasion and therapy resistance [3,4,9,10,11]. Thus, various techniques have been developed to elucidate cell–cell interactions in the tumor microenvironment: current experimental approaches provide either high-throughput molecular analysis (cell sorting-based methods, such as flow cytometry and single cell “omics”) or spatial information through imaging (microscopy-based methods, such as immunohistochemistry), with emerging technologies (imaging-based mass spectrometry and Raman microscopy) combining the advantages of these two protocols [12]. Using these procedures, several efforts have also been made to accurately model the tumor microenvironment in vitro and in vivo. In particular, many experiments are still conducted with 2D co-cultures or animal models. However, 2D monolayers do not fully reflect the physiology of the original tissue, since they do not conserve the tissue-specific architecture and mechanical/biochemical signals. On the other hand, mouse models are usually very expensive and associated with ethical issues, and they are not always representative of human-specific events. By contrast, multicellular 3D systems can overcome these limitations by properly reproducing cell polarity and shape, tissue stiffness and cell–cell/cell-ECM interactions [13,14]. As such, they have shown promise not only in cancer modeling but also in drug discovery and testing [15,16,17]. This review summarizes the main characteristics of the 3D models currently employed in the study of tumor–stroma communication: the pros and cons of each technique are discussed, followed by an extensive overview of the major findings obtained through these methods in the field of cancer research. In particular, due to the increasing interest of the scientific community in the characterization of tumor microenvironment, the present manuscript aims at offering a selection of recent literature illustrating novel approaches for the production and use of 3D cell cultures in the analysis of tumor interactions with ECM, blood vessels, mesenchymal cells and immune system.

## 2. Models of 3D Cell Culture

**Figure 1:**
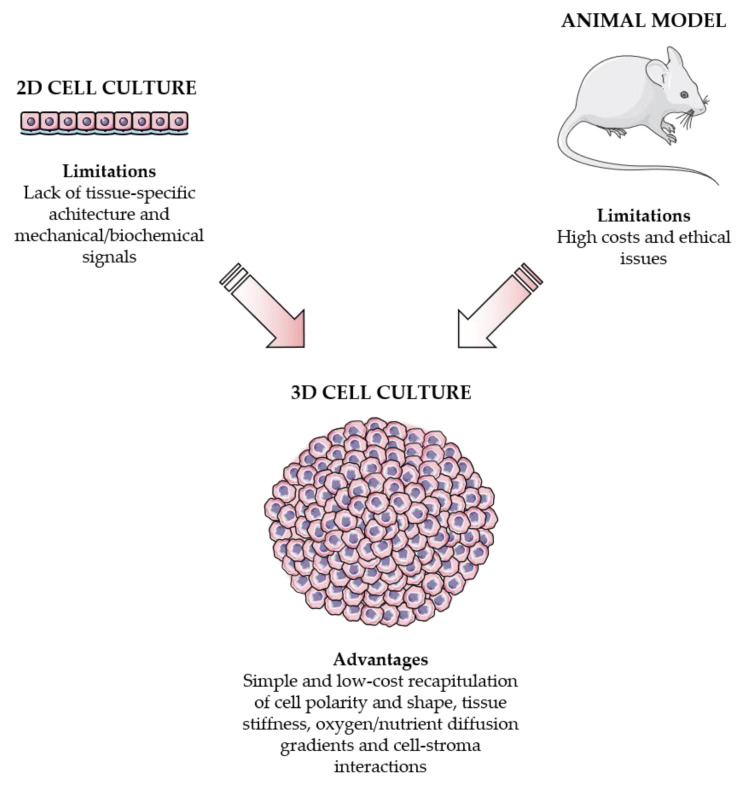
2D cell cultures and animal models vs. 3D cell cultures. On one hand, 2D monolayers do not retain the tissue-specific structure and mechanical/biochemical properties. On the other hand, in vivo models are very expensive and associated with ethical issues. Importantly, 3D systems can overcome these limitations by accurately reproducing cell polarity and shape, tissue stiffness, oxygen/nutrient diffusion gradients and cell-stroma communication.

Although 2D systems have provided an invaluable tool to investigate the molecular bases of tumor biology and promote the preclinical development of many anti-cancer drugs over the years, the limits of this technology are evident [13,14]. As mentioned above, the complexity of tumor microenvironment, composed of malignant cells and stroma, is far too intricate to be reproduced in a 2D monolayer. The best option today available is represented by in vivo models; however, they cannot always be utilized or afforded by researchers all over the world, due to ethical and cost reasons. Therefore, efforts have been made to conceive in vitro techniques able to fill the gap between 2D cultures and animal models and to recreate the most relevant features of a proliferating tumor [18]. 3D culture methods represent an interesting solution to this scientific need, allowing intra-tumor and tumor—stroma contacts and, thus, closely resembling a real tumor mass growing in a living organism. Moreover, unlike 2D settings, in a 3D arrangement cancer cells are not homogeneously exposed to nutrients and oxygen, and thereby not all the neoplastic cells can receive an adequate amount of energy supply, a condition causing important biological consequences (e.g., cancer growth in starvation/hypoxic conditions). Similarly, drugs may not be able to permeate the entire cell culture, making data obtained through 3D systems more predictive of the anti-tumor activity of bioactive molecules. Finally, 3D technologies are characterized by an increased stability and longer lifespans than standard cultures and are thus more suitable for experiments that require the evaluation of tumor cell responses to different stimuli over a long period of time [16,19] (Figure 1).

Historically, one of the first examples of 3D approach is represented by ex vivo culture of whole organs or tissue explants. Although this method can reproduce the complexity and the biology of native tissues, sample viability is limited, and results obtained using these protocols are often difficult to interpret. Hence, alternative techniques have been developed to overcome the issues of utilizing fresh tumor specimens, without losing the possibility to work in 3D arrangements [19,20].

To date, several protocols are available to grow tumor cells in a 3D setting, and they can be generally classified in two broad groups, defined as scaffold- and non-scaffold-based approaches [21].

Scaffold-based techniques require the use of an artificial support that mimics the ECM, providing an anchorage for cancer cells. Once attached to the support, cells can proliferate and migrate across its interstices, acquiring the typical hallmarks of an in vivo tumor. These scaffolds are mainly designed by using natural (i.e., collagen, hyaluronic acid and gelatin) or synthetic (i.e., polycaprolactone, polyglycolide and polylactide) polymers, to produce acellular matrices or hydrogels in which tumor cells can be seeded or dispersed. Natural polymers display higher biocompatibility and lower toxicity, while the synthetic materials exhibit improved workability and versatility, being easier to process. In both cases, the porosity of the obtained substrate allows oxygen, nutrients and drugs to reach the proliferating cells and also facilitates the removal of waste molecules, thus providing appropriate cell culture conditions [22]. However, although the physicochemical properties of these structures can be optimized to obtain an environment that resembles that of the tumor stroma and suits the experimental requirements for hypoxia- or drug-based studies, the use of either natural or synthetic materials may present some disadvantages. For instance, polymers isolated from natural sources may have quite different composition, affecting the reproducibility of the experiments, while the characteristics of synthetic compounds are not always superimposable with those of the ECM [19,23,24].

On the contrary, the only ECM components that are present in non-scaffold-based platforms are those produced by tumor cells themselves, that can aggregate forming the so-called tumor spheroids. Therefore, irrespective of the protocol chosen, the main goal of this method is to promote cancer cell self-assembly, maximizing cell–cell interaction, adhesion and, eventually, aggregation. In this regard, there are different options to achieve spheroid formation, extensively detailed in [25]. Briefly, in agitation-based techniques, cancer cells are cultured in specific devices (i.e., spinner flasks, rotating wall vessels, roller tubes and gyratory shakers), where they are maintained in suspension by applying a constant stirring or rotation that prevent the adhesion to the culture system and, at the same time, induce tumoroid self-assembly. On the other hand, it is also possible to obtain spheroids by static methods. In this case, culture plates are usually coated with materials, such as agar or agarose, that impede tumor cell attachment to plate surface, facilitating cell aggregation. Likewise, hanging-drop techniques allow spheroid generation in the form of droplets: cancer cell suspensions are dispensed into the wells of a mini-tray that is subsequently reversed upside down, promoting tumoroid generation through the simultaneous action of surface tension and gravity. Notably, the dimensions of the spheroids can be controlled by changing the diameter of the droplets [26,27,28,29].

Finally, a new example of 3D technology is represented by the multichannel microfluidic perfusion culture system, also known as “organ-on-a-chip”. This technique is based on the use of microfluidic devices that can be made by plastic, glass or synthetic polymers. The system is constituted by separated compartments incorporated with different cell types, including endothelial and mesenchymal cells, in the presence or absence of ECM. By maintaining the architecture of living tissues, these tools can be utilized to study the interplay between cancer cells and the surrounding stroma [30,31].

The key features of current in vitro 3D models are presented in Table 1.

## 3. Dissecting Tumor–Stroma Interactions in 3D Models

**Figure 2:**
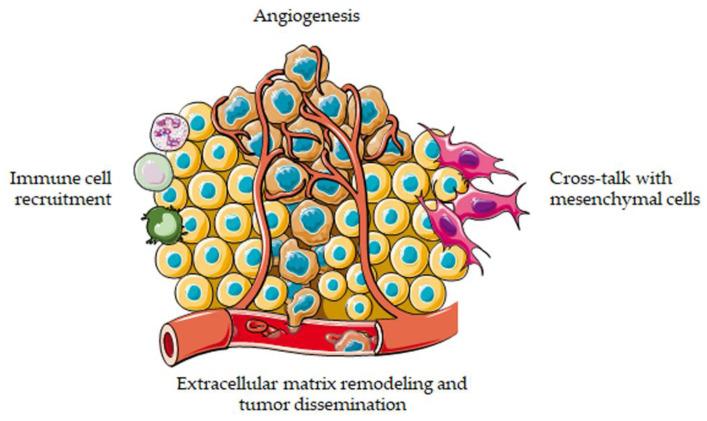
3D cell cultures to model the tumor microenvironment. 3D systems can be employed to study the interactions of cancer cells with extracellular matrix, blood vessels, mesenchymal cells and immune system.

As described above, 3D cell cultures have recently emerged as promising tools to decipher tumor–stroma interactions and to assess the efficacy of anti-cancer agents in vitro. Indeed, an increasing number of studies has pointed out the accuracy and reliability of these approaches in recreating the original tumor microenvironment and in predicting in vivo drug response [10,11,12]. The main results achieved in cancer biology and therapy by applying these models are summarized in the following paragraphs (Figure 2).

### 3.1. Interactions with Extracellular Matrix

Cancer cell behavior is strongly influenced by the characteristics of the extracellular compartment, and qualitative/quantitative modifications of ECM play a key role in determining cancer cell phenotype. Since topological/mechanical properties of the ECM are hard to study in vivo, defined biomimetic in vitro models are needed. In this regard, it has been shown that breast cancer cell morphology, cluster formation and invasion are finely regulated by the fibril diameter of 3D collagen networks [32]. Similarly, a recent study by Seo et al. suggests that an increase in collagen fiber thickness promotes the differentiation of human adipocyte stem cells into myofibroblasts [33]. These results are in line with the obesity-dependent pro-tumor changes observed in the mechanics of interstitial ECM of breast cancer [34].

Collagen I is a fundamental component of breast architecture, providing support to the underlying epithelium. The interactions between this protein and cell surface integrins are not only involved in normal mammary gland function, but also in tumorigenesis [35]. Indeed, it has been demonstrated that MDA-MB-231 cells tend to migrate from a dense to a loose collagen network, showing a preference for a specific 3D matrix composition [36,37]. More importantly, collagen density has been reported to drive a metabolic shift in breast cancer cells, promoting a rapid transition towards a glutamine-dependent phenotype [38]. In this setting, it should also be outlined that an increase in ECM density can enhance the secretion of pro-inflammatory cytokines by M1 macrophages, while ECM stiffening triggers a switch from M1 to M2 polarization [39,40]. In addition, collagen fibrils can regulate the activity of tumor-infiltrating T cells [41], indicating that matrix composition can affect the behavior of various cells in the tumor stroma.

Hyaluronan (HA) is known to be implicated in cancer cell dissemination [42]. Interestingly, the molecular weight of HA can influence tissue mechanics both in vitro and in vivo, modulating melanoma cell proliferation and invasion within 3D ECM [43]. A similar effect has been reported in mammary cancer cells, where treatment with 200 kDa HA resulted in the upregulation of Ε-cadherin and downregulation of HA synthase 2, fibronectin, slug and snail [44]. A recent study by Sapudom et al. has also evidenced the distinct behavior of normal human dermal and activated pro-tumor fibroblasts in terms of HA synthesis and 3D matrix remodeling, pointing to a specific impact of tumor cell invasion [45].

Metastatic dissemination of cancer cells allows the tumor mass to invade the surrounding tissues and eventually enter the blood and lymphatic circulation. Cancer cell invasion is influenced by the communication with the tumor microenvironment and is accompanied by ECM degradation by matrix metalloproteinases (MMPs) [46]. Indeed, several studies have demonstrated the crucial role of MMPs in promoting tumor progression and have proposed these proteins as suitable targets for cancer therapy [47]. In this context, 3D cell cultures offer a reliable experimental setting to unravel the complex mechanisms associated with ECM remodeling in tumor invasion and to test new anti-cancer agents, such as MMP inhibitors [48,49,50,51,52,53,54,55]. As shown above, the analysis of the interactions between tumor cells and ECM is generally performed by culturing cell spheroids in embedding gels containing ECM components, such as collagen [56,57,58]. Remarkably, a novel 3D system composed of a synthetic hydrogel matrix incorporating biomimetic cell integrin-binding motifs (e.g., RGD peptides) and possessing the ability of being degraded by cell-secreted MMPs has been recently used to study ovarian cancer cell behavior. Interestingly, in these experimental conditions tumor cell proliferation was observed only after integrin engagement and proteolytic remodeling of the ECM in the surrounding microenvironment. Moreover, due to its flexibility and long-term stability, this 3D setting was found to represent an effective culture system to screen different anti-cancer drugs and assess the development of chemoresistance [59]. Similarly, a rapid and low-cost CO_2_ laser ablation-based method for concave microwell creation from a conventional untreated culture dish has been proposed to generate hydrogel-embedded multicellular aggregates for pancreatic cancer cell migratory phenotype characterization and preclinical anti-tumor drug screening [60].

Fibroblasts are the main effector cells in tissue formation, being able to synthesize ECM. Therefore, a growing number of studies has focused on the analysis of their role in determining tumor cell migration [61,62,63]. In particular, in a new 3D model developed by Zhang et al. different cancer cell types (CaKi-1, HeLa, A375 and A549) and fibroblasts were mixed in a 50% Matrigel suspension [64]; droplets of the mixture were then seeded, generating an ECM-like network in which migrating cells can be visualized and monitored by time lapse microscopy [64]. Likewise, a spheroid invasion assay was established to assess the migration pattern of co-cultured fibroblasts and epithelial cancer cells in organotypic ECM. Notably, the possibility to track the behavior of both cell types over a period of 24–72 h was pivotal to verify the involvement of fibroblasts in the collective invasion of tumor cells. Indeed, fibroblasts were demonstrated to be located at the leading front within the invading cell strands and to trigger cancer invasion through specific cell–cell contacts similar to those observed in human tumor tissues [65].

A dense stroma containing ECM components is typical of pancreatic ductal adenocarcinoma (PDAC). A multichannel microfluidic chip has been exploited to characterize the migratory potential of various PDAC cells differing from each other for their phenotype. Intriguingly, PANC-1 cells, possessing mesenchymal features, were shown to undergo individual invadopodium-mediated migration, correlated with upregulation of MMPs and collagen cleavage. By contrast, BxPC-3 cells, having an epithelial phenotype, exhibited a proteolysis-independent heterotypic migration, resulting from the combination of collective and individual cell invasion in either mesenchymal or amoeboid mode [66].

### 3.2. Interactions with Endothelial Cells

Angiogenesis, the process responsible for new blood vessel generation from pre-existing vasculature, is one of the main steps in cancer initiation and progression [67]. Although easy-to-perform and low-cost tube formation assays are commonly utilized to study this mechanism, they are insufficient to address the complexity of tumor interactions with capillaries [68]. To recapitulate this crosstalk, Ghiabi et al. have developed vascularized tumoroids using different breast carcinoma cell lines, demonstrating that endothelial cells can directly promote cancer proliferation, stemness and invasiveness via Notch activation [69]. Parallelly, they observed that the stroma itself could also benefit from this dialog by undergoing endothelial-to-mesenchymal transition (EndMT). Overall, these phenotypic changes resulted in a higher tumor aggressiveness in vivo [70].

Chiew et al. have also used a 3D platform to analyze the communication between hepatocellular carcinoma and small vessels [71]. In their model, a complex capillary network was formed inside a spheroid exhibiting a hypoxic central region resembling that of a real tumor mass. Notably, they evaluated the cytotoxicity of sorafenib, sunitinib and axitinib on endothelial cells and found that the hypoxic core could significantly modulate drug uptake, leading to a more realistic penetration gradient relative to 2D monolayers [71]. These data were also confirmed in a vascularized ovarian cancer model, in which tumor cells were able to confer bevacizumab resistance to the endothelium [72].

Epithelial-to-mesenchymal transition (EMT) plays a crucial role in the early stages of cancer cell dissemination, leading to metastasis formation [73]. Strategies based on EMT prevention could then improve the outcome of advanced stage malignancies [74]. However, current assays only examine the dispersion of isolated cancer cells in conventional 2D microwell systems, an approach that fails to account for the essential interactions that occur with other nearby cell types, including endothelial cells, in the 3D tumor microenvironment. In this context, two novel microfluidic systems integrating tumor cell spheroids in a 3D hydrogel scaffold in close co-culture with an endothelial monolayer have been designed. Intriguingly, significant differences in response to EMT-targeting drugs have been highlighted between 2D and 3D settings and between mono- and co-cultures [75,76].

Another layer of complexity has been added by Ehsan et al., who generated multicellular spheroids containing endothelial tubes and cancer cells from breast, lung or colon, co-embedded with isolated fibroblasts [77]. Intriguingly, this system mimics the specific angiogenic abilities of each tumor, with breast cancer cells exhibiting more capillaries compared to lung or colon carcinoma. Moreover, tumor cell-specific intravasation has been observed, as colorectal cancer cells were shown to easily localize and migrate through the vessels, especially in low-oxygen conditions [77].

More recently, the interactions between prostate cancer and capillaries have been investigated in a triple co-culture with mesenchymal stem cells (MSCs). This protocol recapitulates the first avascular growing step of the tumor mass, with endothelial tubes only establishing contacts at the tumoroid surface. Interestingly, tumor cells cultured within this system were less responsive to chemotherapy with respect to 2D monolayers and displayed a proliferation rate closer to that of the in vivo prostate cancer microenvironment [78].

The above 3D co-culture techniques can be utilized to dissect the crosstalk between tumor cells and blood vessels, without including lymphatic structures. To analyze the molecular mechanisms of intravasation in both blood and lymphatic capillaries, pancreatic and colorectal cancer cell lines derived from hematogenous or lymphogenous metastasis have been employed in a novel model of tumor invasion, in which the specificity of these cells for each type of vessel was successfully reproduced. Notably, it has been shown that cancer cells were characterized by distinct MMP secretion profiles according to their target vasculature, highlighting the importance of this technique in mimicking the tumor-specific process of metastatic colonization [79]. Similarly, a skin construct composed of melanoma cells and a blood/lymphatic capillary network has been recently engineered, not only recapitulating the first steps of tumor dissemination but also highlighting an effect of the microenvironment on vemurafenib resistance [80].

### 3.3. Interactions with Mesenchymal Cells

Cancer-associated fibroblasts (CAFs) and adipocytes (CAAs), as well as bone marrow MSCs are fundamental constituents of tumor stroma and have been increasingly acknowledged as major contributors to cancer initiation and progression. Thereby, in recent years, several studies have focused on the dissection of the crosstalk between these cell types and tumor cells [81,82,83].

When it comes to co-cultures, one of the main challenges is to keep a ratio between the co-seeded cell types that reflects the in vivo setting, considering the proliferation rate of each component. In a recent work by Eder et al., prostate cancer cells were co-cultured with CAFs: an increased number of tumor cells compared to fibroblasts was found within the spheroids, mimicking what generally occurs in mice [84]. The consistency of 3D systems with the in vivo microenvironment was also confirmed by Brancato et al., who recreated a PDAC microtissue where tumor cells were shown to be the predominant portion of proliferating cells with respect to mesenchymal cells [85]. Likewise, 3D co-cultures of different colon, breast and lung cancer cell lines with CAFs displayed the same growth dynamics and hypoxia gradients of native tumors, retaining the architectural features of the original tumor mass [86,87]. Collectively, these data show how 3D models can be used to avoid the artefact of a non-representative ratio between different cell types usually observed in 2D monolayers.

It is known that mesenchymal cells can directly promote cancer cell proliferation and migration, and the use of 3D co-cultures has largely supported this evidence. Indeed, CAFs have been reported to promote tumor growth and invasion in several 3D settings, by secreting different soluble factors and MMPs [88,89,90,91,92,93,94,95]. Similarly, human adipose tissue has been shown to enhance breast cancer cell migratory potential in a collagen matrix-based 3D system, while MSCs have been found to trigger Akt activation and rewire glucose metabolism in a 3D model of the ER+ mammary carcinoma bone marrow niche [96,97]. Intriguingly, the above dialog appears to be bidirectional: different tumor cell lines grown in 3D have been demonstrated to modulate fibroblast proliferation and motility, leading to stromal desmoplasia [98,99].

Pancreatic stellate cells (PSCs) represent a major component of PDAC microenvironment. Notably, culturing this cell type in 3D co-spheroids has proved to be a useful method to study cell–cell interactions. Indeed, clear evidence of mutual influence has been observed, with pancreatic cancer cells exhibiting enhanced growth and expression of EMT markers and with PSCs switching toward a myofibroblast phenotype [100,101,102,103].

3D co-cultures have also allowed the dissection of more complex phenomena. For example, Bersini et al. have recently engineered a tri-culture system aimed at deciphering the mechanisms of breast cancer metastasis following extravasation to bone [104]. In this model, highly metastatic MDA-MB-231 cells were free to migrate through an endothelial monolayer and reach collagen-embedded osteo-differentiated MSCs. Notably, both extravasation and migration to the bone marrow niche were significantly higher in the osteo-cell-conditioned microenvironment relative to collagen gel-only matrices, and the CXCL5-CXCR2 signaling was found to play a key role in these processes [104]. Parallelly, MCF10A cells were co-seeded with fibroblasts and human adipose-derived stem cells in 3D and were demonstrated to express higher levels of casein mRNA, a marker of mammary cell differentiation, with respect to breast cancer cells in monoculture or in co-culture with either cell type [105].

As obvious consequence of the above observations, 3D platforms can be utilized to test anti-cancer drug response in more physiologically relevant contexts. In this regard, Houshmand et al. developed a realistic melanoma model composed of tumor cells and fibroblasts, highlighting the ability of the latter to modulate cancer sensitivity to vemurafenib through secretion of various proteins, such as interleukin-6 (IL-6), IL-8, hepatocyte growth factor (HGF) and transforming growth factor (TGF) [106]. Similarly, secretome profiling of heterotypic spheroids evidenced the role of CAFs in conferring HIF-1-mediated photodynamic resistance to colorectal carcinoma cells [107,108]. In addition, coculturing of neuroblastoma and fibroblast cells in 3D resulted in cyclooxygenase/mPGES-1/PGE_2_ -driven chemoresistance [109]. Moreover, in a leukemic bone marrow niche-like setting higher tolerance to azacytidine and cytarabine was observed, due to Bcl-2 overexpression [110]. Likewise, prostate cancer cells co-seeded with MSCs were able to survive to hormonal therapy via paracrine IL-6-associated decrease in hormone receptor expression, which was restored through inhibition of IL-6 or JAK pathways. Conversely, breast tumoroids retained ER expression in the presence of mesenchymal cells but acquired drug resistance via an IL-6-independent PI3K and ERK activation [111]. Finally, a more complex 3D hepatocyte chip was employed to predict in vivo drug hepatotoxicity: the IC50 values of five different drugs were evaluated and were confirmed to be comparable to their LD50 values in mice [112].

### 3.4. Interactions with Immune Cells

The interactions between tumor and immune cells are highly complex. The physiological role of the immune system is to find and eliminate cancer cells; however, according to the “3E’s rule”, the “Elimination” step is followed by the “Equilibrium” and “Escape” phases. In the equilibrium stage a balance between immune and tumor cells is reached, while the escaping phase is due to the ability of cancer cells to overcome the attack of the immune system, both by reducing their own immunogenicity and by turning the immune components toward a pro-tumor phenotype [113,114]. Adding complexity to these phenomena, the immune system is composed of different cell types having distinct functions and interacting with each other, eventually orchestrating the resulting anti-tumor or pro-tumor effects [113,115]. The molecular mechanisms underlying these processes are not fully elucidated yet. However, their understanding is crucial to highlight new molecular targets and to specifically design immune cell-based therapies. Nowadays, the gold standard model for studying the immune landscape of human tumors is the immune-competent animal model, that can reflect the complex networking between the various cell types within the tumor microenvironment, as well as their recruitment from lymphoid/myeloid organs. The obvious limitation of this method relies on the use of syngeneic animals bearing non-human tumors. Conversely, human cancer cells can be xenotransplanted in immune-deficient mice, completely losing the contribution of the immune system to tumor development and progression. Elegant, humanized mouse models have been recently developed; however, they are highly expensive and could fail to predict what generally occurs in the human body [116,117,118]. Thus, several efforts are ongoing to recapitulate the human tumor-immune system communication in vitro. This modeling must take into account that different tumors are characterized by distinct immune signatures: (1) inflamed (or “hot”) tumors are infiltrated with immune cells, interacting with them through direct contact (i.e., PD1/PDL1 interaction); (2) immune-excluded cancers are surrounded by immune cells, that are in turn confined to the borders of the tumor mass and are not able to infiltrate it; (3) finally, in immune-desert (or “cold”) malignancies, immune cells are completely unable to reach the tumor tissue, so that it is nor surrounded nor infiltrated by immune cells [113]. In this context, 3D settings have been reported to provide a more reliable model of immune cell infiltration and activation [119]. For instance, they have been employed to dissect the role of Treg cells in different gastric cancer subtypes, demonstrating that immune cells are indeed able to actively infiltrate intestinal-type gastric cancer spheroids while accumulating at the peripheral sites of diffusive-type gastric cancer spheroids, thus highlighting that their penetration ability depends on the histological features of the tumor [120]. Similarly, a recent study by Majedi et al. has shown that T cell activation and migration are positively modulated by stiffer rather than softer matrices, indicating that the choice of the 3D matrix to be used in cancer modeling must be made according to the tumor microenvironment that needs to be recapitulated [121].

Another important aspect to be considered when reproducing the tumor-immune system interactions is the recruitment of the immune cells itself: apart from few resident cells (i.e., tissue-resident macrophages), immune components are actively engaged within the tumor mass. Then, the presence of a complex 3D environment could be highly predictive of anti-cancer immunotherapy success, not only for those approaches aimed at blocking pro-tumor infiltrating immune cells, but also for those strategies designed to turn a cold tumor into a hot malignancy suitable for immune-checkpoint inhibition treatment [119]. For example, in a recent study by Courau et al. 3D cell cultures were utilized to investigate the crosstalk between colon cancer and NK/T cells, as well as the potential of new immunomodulatory therapies [122]. To produce a clinically relevant functional assay, the authors generated an allogenic model, composed of a colorectal carcinoma cell line co-cultured with immune cells from healthy donors, and adapted it to a novel autologous culture, based on patient-derived spheroids co-seeded with immune cells from the same patient, demonstrating that NK and T cells can invade and eradicate the tumor mass via NKG2D. However, the presence of anti-tumor immune cells led to the upregulation of inhibitory molecules, namely HLA-E, in cancer cells, suggesting that the latter can evade the tumor-suppressing immune response through NKG2A/HLA-E binding. Given these observations, the authors exploited these experimental models to successfully test the efficacy of a novel therapeutic strategy, combining anti-MICA/B antibodies (thus increasing the immune-mediated cytotoxic effects) and an anti-NKG2A antibody (thus avoiding tumor immune-escape), showing a relevant synergistic action [122].

Co-culturing tumor and immune cells in a 3D environment has also shown promise in the study of specific processes such as cancer cell collective migration, frequently occurring in ovarian carcinoma. In this regard, Surendran et al. have recently developed a 3D tumor/neutrophils-on-a-chip device in which ovarian cancer spheroids elicit an in vivo-like immune response associated with neutrophil chemotaxis and extracellular trap release, that in turn correlates with ovarian cancer cell collective invasion [123].

Other studies have demonstrated that a 3D setting better recapitulates the phenotype of both cancer and immune cells, as it occurs in patients. In particular, a complex 3D-bioprinted glioblastoma tetra-culture model has been found to be a relevant tool to assess macrophage polarization toward an M2 phenotype. Moreover, this technology faithfully reproduced the patients’ transcriptional profile [124]. It has also been demonstrated that 3D cultures are more reliable than 2D standard bilayers in terms of expression of immune-checkpoint molecules, with B7-H3, PDL2 and PVR levels being significantly different between the two experimental conditions. Thus, the choice of the model might importantly affect immunotherapy testing [125].

Like other treatment regimens, the response to immune-checkpoint inhibitors is strictly dependent on the single patient. Consequently, the selection of the best therapeutic strategy might benefit of ex vivo screening platforms which involve patient-derived cultures. Appleton et al. have recently engineered an ex vivo model based on the dissociation of patients’ biopsies and their subsequent reconstruction in a 3D culture [126]. This approach has been found to be highly predictive of the changes occurring in the immune composition of ovarian cancer microenvironment, thus representing a relevant tool to identify the best therapeutic option [126].

When considering immune cells, adaptive immunity responses are harder to reproduce in vitro, due to the need of autologous models. To study this type of interactions, it is possible to specifically culture patient-derived biopsies in a 3D setting. Indeed, this model allows to retain the original tumor microenvironment cell composition [113]. Another interesting approach resides on the isolation of T cells from patients’ blood and subsequent seeding in the ex vivo cultured biopsy. Both these approaches are driving cancer research much closer to personalized medicine: importantly, they can be efficiently exploited for testing and customizing CAR-T therapies.

## 4. Conclusions

It is now well established that cancer cells can turn their surrounding stroma into a hospitable home that better meets their growth needs. In response to hostile conditions, such as hypoxia and nutrient deprivation, tumor cells can recruit their neighboring non-malignant cells, including endothelial, mesenchymal and immune cells, as well as the non-cellular components of ECM, for their own benefits. Thereby, disrupting tumor–stroma interactions can be exploited as a novel strategy for future cancer therapy regimens. However, extensive research is still required to understand the complex communication network occurring within the tumor microenvironment and to test the potential of novel drugs in suitable preclinical settings. In this context, in vitro cell cultures represent an indispensable tool to decipher fundamental tumorigenic processes and to identify new cancer therapeutics. Although 2D monolayers have been widely used in cancer research, new 3D systems with more realistic biochemical and biomechanical properties have gained increasing interest by the scientific community. In particular, advances in bioengineering have encouraged the rapid development of easy and economical 3D technologies. Specifically, 3D co-culture models with tumor and stromal cells have been recently obtained, demonstrating great potential in mimicking the cancer niche and the interactions between the tumor and its microenvironment. As such, 3D models can be utilized not only for disease simulation but also for drug discovery and screening. In the future, they are expected to be largely used to confirm preclinical findings, supporting in vivo experimentation and eventually promoting its replacement.

## Figures and Tables

**Table 1:** 3D cell culture techniques.

Technique	Pros	Cons	Ref.
Scaffolds	Accurate tissue recapitulation	Expensive, variability in polymer composition	[14,17,18]
Agitation-based methods	Easy to perform, inexpensive, appropriate for multicellular spheroid generation	Variability in spheroid size, extracellular matrix not addable, inappropriate for migration assays	[19,20,21,22]
Suspension cell cultures	Easy to perform, inexpensive, appropriate for multicellular spheroid generation	Variability in spheroid size, extracellular matrix not addable, inappropriate for migration assays	[19,20,21,22]
Hanging drop	Inexpensive, spheroid size uniformity	Difficult to perform, extracellular matrix not addable, inappropriate for migration assays	[19,20,21,22]
Organ-on-a-chip	Rapid spheroid formation, spheroid size uniformity, constant perfusion and distribution of oxygen and nutrients	Expensive, difficult to perform, specialized equipment and expertise	[23,24]

## References

[1] Tlsty T.D., Coussens L.M. (2006). Tumor stroma and regulation of cancer development. Annu. Rev. Pathol. Mech. Dis..

[2] Pietras K., Östman A. (2010). Hallmarks of cancer: Interactions with the tumor stroma. Exp. Cell Res..

[3] Witz I.P. (2008). Tumor–Microenvironment Interactions: Dangerous Liaisons. Adv. Cancer Res..

[4] Najafi M., Goradel N.H., Farhood B., Salehi E., Solhjoo S., Toolee H., Kharazinejad E., Mortezaee K. (2019). Tumor microenvironment: Interactions and therapy. J. Cell. Physiol..

[5] Zhang X. (2010). Growth factors in tumor microenvironment. Front. Biosci..

[6] Landskron G., De la Fuente M., Thuwajit P., Thuwajit C., Hermoso M.A. (2014). Chronic Inflammation and Cytokines in the Tumor Microenvironment. J. Immunol. Res..

[7] Tao S.-C., Guo S.-C. (2020). Role of extracellular vesicles in tumour microenvironment. Cell Commun. Signal..

[8] Pan Z., Tian Y., Niu G., Cao C. (2019). Role of microRNAs in remodeling the tumor microenvironment (Review). Int. J. Oncol..

[9] McMillin D.W., Negri J.M., Mitsiades C.S. (2013). The role of tumour–stromal interactions in modifying drug response: Challenges and opportunities. Nat. Rev. Drug Discov..

[10] Valkenburg K.C., de Groot A.E., Pienta K.J. (2018). Targeting the tumour stroma to improve cancer therapy. Nat. Rev. Clin. Oncol..

[11] Fontana F., Carollo E., Melling G.E., Carter D.R.F. (2021). Extracellular Vesicles: Emerging Modulators of Cancer Drug Resistance. Cancers.

[12] Nishida-Aoki N., Gujral T.S. (2019). Emerging approaches to study cell-cell interactions in tumor microenvironment. Oncotarget.

[13] Duval K., Grover H., Han L.-H., Mou Y., Pegoraro A.F., Fredberg J., Chen Z. (2017). Modeling Physiological Events in 2D vs. 3D Cell Culture. Physiology.

[14] Jensen C., Teng Y. (2020). Is It Time to Start Transitioning From 2D to 3D Cell Culture?. Front. Mol. Biosci..

[15] Asghar W., El Assal R., Shafiee H., Pitteri S., Paulmurugan R., Demirci U. (2015). Engineering cancer microenvironments for in vitro 3-D tumor models. Mater. Today.

[16] Hoarau-Véchot J., Rafii A., Touboul C., Pasquier J. (2018). Halfway between 2D and Animal Models: Are 3D Cultures the Ideal Tool to Study Cancer-Microenvironment Interactions?. Int. J. Mol. Sci..

[17] Fontana F., Raimondi M., Marzagalli M., Sommariva M., Gagliano N., Limonta P. (2020). Three-Dimensional Cell Cultures as an In Vitro Tool for Prostate Cancer Modeling and Drug Discovery. Int. J. Mol. Sci..

[18] Kapałczyńska M., Kolenda T., Przybyła W., Zajączkowska M., Teresiak A., Filas V., Ibbs M., Bliźniak R., Łuczewski Ł., Lamperska K. (2016). 2D and 3D cell cultures—A comparison of different types of cancer cell cultures. Arch. Med. Sci..

[19] Lv D., Hu Z., Lu L., Lu H., Xu X. (2017). Three-dimensional cell culture: A powerful tool in tumor research and drug discovery (Review). Oncol. Lett..

[20] Meijer T.G., Naipal K.A., Jager A., van Gent D.C. (2017). Ex vivo tumor culture systems for functional drug testing and therapy response prediction. Future Sci. OA.

[21] Chaicharoenaudomrung N., Kunhorm P., Noisa P. (2019). Three-dimensional cell culture systems as an in vitro platform for cancer and stem cell modeling. World J. Stem Cells.

[22] Breslin S., O’Driscoll L. (2013). Three-dimensional cell culture: The missing link in drug discovery. Drug Discov. Today.

[23] Nikolova M.P., Chavali M.S. (2019). Recent advances in biomaterials for 3D scaffolds: A review. Bioact. Mater..

[24] Park Y., Huh K.M., Kang S.-W. (2021). Applications of Biomaterials in 3D Cell Culture and Contributions of 3D Cell Culture to Drug Development and Basic Biomedical Research. Int. J. Mol. Sci..

[25] Ryu N.-E., Lee S.-H., Park H. (2019). Spheroid Culture System Methods and Applications for Mesenchymal Stem Cells. Cells.

[26] Bordanaba-Florit G., Madarieta I., Olalde B., Falcón-Pérez J.M., Royo F. (2021). 3D Cell Cultures as Prospective Models to Study Extracellular Vesicles in Cancer. Cancers.

[27] Langhans S.A. (2018). Three-Dimensional in Vitro Cell Culture Models in Drug Discovery and Drug Repositioning. Front. Pharmacol..

[28] Rodrigues J., Heinrich M.A., Teixeira L.M., Prakash J. (2021). 3D In Vitro Model (R)evolution: Unveiling Tumor–Stroma Interactions. Trends Cancer.

[29] Fontana F., Raimondi M., Marzagalli M., Sommariva M., Limonta P., Gagliano N. (2019). Epithelial-To-Mesenchymal Transition Markers and CD44 Isoforms Are Differently Expressed in 2D and 3D Cell Cultures of Prostate Cancer Cells. Cells.

[30] Sun W., Luo Z., Lee J., Kim H.-J., Lee K., Tebon P., Feng Y., Dokmeci M.R., Sengupta S., Khademhosseini A. (2019). Organ-on-a-Chip for Cancer and Immune Organs Modeling. Adv. Healthc. Mater..

[31] Trujillo-de Santiago G., Flores-Garza B.G., Tavares-Negrete J.A., Lara-Mayorga I.M., González-Gamboa I., Zhang Y.S., Rojas-Martínez A., Ortiz-López R., Álvarez M.M. (2019). The Tumor-on-Chip: Recent Advances in the Development of Microfluidic Systems to Recapitulate the Physiology of Solid Tumors. Materials.

[32] Sapudom J., Rubner S., Martin S., Kurth T., Riedel S., Mierke C.T., Pompe T. (2015). The phenotype of cancer cell invasion controlled by fibril diameter and pore size of 3D collagen networks. Biomaterials.

[33] Seo B.R., Chen X., Ling L., Song Y.H., Shimpi A.A., Choi S., Gonzalez J., Sapudom J., Wang K., Eguiluz R.C.A. (2020). Collagen microarchitecture mechanically controls myofibroblast differentiation. Proc. Natl. Acad. Sci. USA.

[34] Seo B.R., Bhardwaj P., Choi S., Gonzalez J., Eguiluz R.C.A., Wang K., Mohanan S., Morris P.G., Du B., Zhou X.K. (2015). Obesity-dependent changes in interstitial ECM mechanics promote breast tumorigenesis. Sci. Transl. Med..

[35] Keely P.J. (2011). Mechanisms by Which the Extracellular Matrix and Integrin Signaling Act to Regulate the Switch Between Tumor Suppression and Tumor Promotion. J. Mammary Gland Biol. Neoplasia.

[36] Bordeleau F., Tang L.N., Reinhart-King C.A. (2013). Topographical guidance of 3D tumor cell migration at an interface of collagen densities. Phys. Biol..

[37] Sapudom J., Rubner S., Martin S., Pompe T. (2016). Mimicking Tissue Boundaries by Sharp Multiparameter Matrix Interfaces. Adv. Healthc. Mater..

[38] Morris B.A., Burkel B., Ponik S.M., Fan J., Condeelis J.S., Aguirre-Ghiso J.A., Castracane J., Denu J.M., Keely P.J. (2016). Collagen Matrix Density Drives the Metabolic Shift in Breast Cancer Cells. EBioMedicine.

[39] Sapudom J., Mohamed W.K.E., Garcia-Sabaté A., Alatoom A., Karaman S., Mahtani N., Teo J.C.M. (2020). Collagen Fibril Density Modulates Macrophage Activation and Cellular Functions during Tissue Repair. Bioengineering.

[40] Friedemann M., Kalbitzer L., Franz S., Moeller S., Schnabelrauch M., Simon J.-C., Pompe T., Franke K. (2017). Instructing Human Macrophage Polarization by Stiffness and Glycosaminoglycan Functionalization in 3D Collagen Networks. Adv. Healthc. Mater..

[41] Kuczek D.E., Larsen A.M.H., Thorseth M.-L., Carretta M., Kalvisa A., Siersbæk M.S., Simões A.M.C., Roslind A., Engelholm L.H., Noessner E. (2019). Collagen density regulates the activity of tumor-infiltrating T cells. J. Immunother. Cancer.

[42] Lokeshwar V.B., Mirza S., Jordan A. (2014). Targeting Hyaluronic Acid Family for Cancer Chemoprevention and Therapy. Adv. Cancer Res..

[43] Sapudom J., Ullm F., Martin S., Kalbitzer L., Naab J., Möller S., Schnabelrauch M., Anderegg U., Schmidt S., Pompe T. (2017). Molecular weight specific impact of soluble and immobilized hyaluronan on CD44 expressing melanoma cells in 3D collagen matrices. Acta Biomater..

[44] Tavianatou A.-G., Piperigkou Z., Barbera C., Beninatto R., Masola V., Caon I., Onisto M., Franchi M., Galesso D., Karamanos N.K. (2019). Molecular size-dependent specificity of hyaluronan on functional properties, morphology and matrix composition of mammary cancer cells. Matrix Biol. Plus.

[45] Sapudom J., Müller C.D., Nguyen K.-T., Martin S., Anderegg U., Pompe T. (2020). Matrix Remodeling and Hyaluronan Production by Myofibroblasts and Cancer-Associated Fibroblasts in 3D Collagen Matrices. Gels.

[46] Cabral-Pacheco G.A., Garza-Veloz I., La Rosa C.C.-D., Ramirez-Acuña J.M., Perez-Romero B.A., Guerrero-Rodriguez J.F., Martinez-Avila N., Martinez-Fierro M.L. (2020). The Roles of Matrix Metalloproteinases and Their Inhibitors in Human Diseases. Int. J. Mol. Sci..

[47] Levin M., Udi Y., Solomonov I., Sagi I. (2017). Next generation matrix metalloproteinase inhibitors—Novel strategies bring new prospects. Biochim. Biophys. Acta Mol. Cell Res..

[48] Liu W., Li L., Wang X., Ren L., Wang X., Wang J., Tu Q., Huang X., Wang J. (2010). An integrated microfluidic system for studying cell-microenvironmental interactions versatilely and dynamically. Lab Chip.

[49] Sung K.E., Yang N., Pehlke C., Keely P.J., Eliceiri K.W., Friedl A., Beebe D.J. (2011). Transition to invasion in breast cancer: A microfluidic in vitro model enables examination of spatial and temporal effects. Integr. Biol..

[50] Koch T.M., Münster S., Bonakdar N., Butler J.P., Fabry B. (2012). 3D Traction Forces in Cancer Cell Invasion. PLoS ONE.

[51] Shen Y.-I., Abaci H.E., Krupski Y., Weng L.-C., Burdick J.A., Gerecht S. (2014). Hyaluronic acid hydrogel stiffness and oxygen tension affect cancer cell fate and endothelial sprouting. Biomater. Sci..

[52] Mosadegh B., Lockett M.R., Minn K.T., Simon K.A., Gilbert K., Hillier S., Newsome D., Li H., Hall A.B., Boucher D.M. (2015). A paper-based invasion assay: Assessing chemotaxis of cancer cells in gradients of oxygen. Biomaterials.

[53] Yamada K.M., Sixt M. (2019). Mechanisms of 3D cell migration. Nat. Rev. Mol. Cell Biol..

[54] Doyle A.D., Yamada K.M. (2016). Mechanosensing via cell-matrix adhesions in 3D microenvironments. Exp. Cell Res..

[55] Antoni D., Burckel H., Josset E., Noel G. (2015). Three-Dimensional Cell Culture: A Breakthrough in Vivo. Int. J. Mol. Sci..

[56] Tamaki M., McDonald W., Amberger V.R., Moore E., Del Maestro R.F. (1997). Implantation of C6 astrocytoma spheroid into collagen type I gels: Invasive, proliferative, and enzymatic characterizations. J. Neurosurg..

[57] Lakka S.S., Gondi C.S., Yanamandra N., Olivero W.C., Dinh D.H., Gujrati M., Rao J.S. (2004). Inhibition of cathepsin B and MMP-9 gene expression in glioblastoma cell line via RNA interference reduces tumor cell invasion, tumor growth and angiogenesis. Oncogene.

[58] Ilina O., Bakker G.-J., Vasaturo A., Hofmann R.M., Friedl P. (2011). Two-photon laser-generated microtracks in 3D collagen lattices: Principles of MMP-dependent and -independent collective cancer cell invasion. Phys. Biol..

[59] Loessner D., Stok K.S., Lutolf M.P., Hutmacher D.W., Clements J.A., Rizzi S.C. (2010). Bioengineered 3D platform to explore cell–ECM interactions and drug resistance of epithelial ovarian cancer cells. Biomaterials.

[60] Tu T.-Y., Wang Z., Bai J., Sun W., Peng W.K., Huang R.Y.-J., Thiery J.-P., Kamm R.D. (2014). Rapid Prototyping of Concave Microwells for the Formation of 3D Multicellular Cancer Aggregates for Drug Screening. Adv. Healthc. Mater..

[61] Attieh Y., Clark A.G., Grass C., Richon S., Pocard M., Mariani P., Elkhatib N., Betz T., Gurchenkov B., Vignjevic D.M. (2017). Cancer-associated fibroblasts lead tumor invasion through integrin-β3–dependent fibronectin assembly. J. Cell Biol..

[62] Erdogan B., Ao M., White L.M., Means A.L., Brewer B.M., Yang L., Washington M.K., Shi C., Franco O.E., Weaver A.M. (2017). Cancer-associated fibroblasts promote directional cancer cell migration by aligning fibronectin. J. Cell Biol..

[63] Miyazaki K., Oyanagi J., Hoshino D., Togo S., Kumagai H., Miyagi Y. (2019). Cancer cell migration on elongate protrusions of fibroblasts in collagen matrix. Sci. Rep..

[64] Zhang Y., Jiang B., Lee M.H. (2020). A Novel 3D Model for Visualization and Tracking of Fibroblast-Guided Directional Cancer Cell Migration. Biology.

[65] Labernadie A., Kato T., Brugués A., Serra-Picamal X., Derzsi S., Arwert E., Weston A., González-Tarragó V., Elosegui-Artola A., Albertazzi L. (2017). A mechanically active heterotypic E-cadherin/N-cadherin adhesion enables fibroblasts to drive cancer cell invasion. Nat. Cell Biol..

[66] Kim S.-K., Jang S.D., Kim H., Chung S., Park J.K., Kuh H.-J. (2020). Phenotypic Heterogeneity and Plasticity of Cancer Cell Migration in a Pancreatic Tumor Three-Dimensional Culture Model. Cancers.

[67] Lugano R., Ramachandran M., Dimberg A. (2020). Tumor angiogenesis: Causes, consequences, challenges and opportunities. Cell. Mol. Life Sci..

[68] Brassard-Jollive N., Monnot C., Muller L., Germain S. (2020). In vitro 3D Systems to Model Tumor Angiogenesis and Interactions With Stromal Cells. Front. Cell Dev. Biol..

[69] Ghiabi P., Jiang J., Pasquier J., Maleki M., Abu-Kaoud N., Rafii S., Rafii A. (2014). Endothelial Cells Provide a Notch-Dependent Pro-Tumoral Niche for Enhancing Breast Cancer Survival, Stemness and Pro-Metastatic Properties. PLoS ONE.

[70] Ghiabi P., Jiang J., Pasquier J., Maleki M., Abu-Kaoud N., Halabi N., Guerrouahen B.S., Rafii S., Rafii A. (2015). Breast cancer cells promote a notch-dependent mesenchymal phenotype in endothelial cells participating to a pro-tumoral niche. J. Transl. Med..

[71] Chiew G.G.Y., Wei N., Sultania S., Lim S., Luo K.Q. (2017). Bioengineered three-dimensional co-culture of cancer cells and endothelial cells: A model system for dual analysis of tumor growth and angiogenesis. Biotechnol. Bioeng..

[72] Guerrouahen B.S., Pasquier J., Kaoud N.A., Maleki M., Beauchamp M.-C., Yasmeen A., Ghiabi P., Lis R., Vidal F., Saleh A. (2014). Akt-Activated Endothelium Constitutes the Niche for Residual Disease and Resistance to Bevacizumab in Ovarian Cancer. Mol. Cancer Ther..

[73] Ribatti D., Tamma R., Annese T. (2020). Epithelial-Mesenchymal Transition in Cancer: A Historical Overview. Transl. Oncol..

[74] Davis F.M., Stewart T.A., Thompson E.W., Monteith G.R. (2014). Targeting EMT in cancer: Opportunities for pharmacological intervention. Trends Pharmacol. Sci..

[75] Aref A.R., Huang R.Y.-J., Yu W., Chua K.-N., Sun W., Tu T.-Y., Bai J., Sim W.-J., Zervantonakis I.K., Thiery J.P. (2013). Screening therapeutic EMT blocking agents in a three-dimensional microenvironment. Integr. Biol..

[76] Bai J., Tu T.-Y., Kim C., Thiery J.P., Kamm R.D. (2015). Identification of drugs as single agents or in combination to prevent carcinoma dissemination in a microfluidic 3D environment. Oncotarget.

[77] Ehsan S.M., Welch-Reardon K.M., Waterman M.L., Hughes C.C.W., George S.C. (2014). A three-dimensional in vitro model of tumor cell intravasation. Integr. Biol..

[78] Bray L.J., Binner M., Holzheu A., Friedrichs J., Freudenberg U., Hutmacher D.W., Werner C. (2015). Multi-parametric hydrogels support 3D in vitro bioengineered microenvironment models of tumour angiogenesis. Biomaterials.

[79] Nishiguchi A., Matsusaki M., Kano M.R., Nishihara H., Okano D., Asano Y., Shimoda H., Kishimoto S., Iwai S., Akashi M. (2018). In vitro 3D blood/lymph-vascularized human stromal tissues for preclinical assays of cancer metastasis. Biomaterials.

[80] Bourland J., Fradette J., Auger F.A. (2018). Tissue-engineered 3D melanoma model with blood and lymphatic capillaries for drug development. Sci. Rep..

[81] Shiga K., Hara M., Nagasaki T., Sato T., Takahashi H., Takeyama H. (2015). Cancer-Associated Fibroblasts: Their Characteristics and Their Roles in Tumor Growth. Cancers.

[82] Lengyel E., Makowski L., DiGiovanni J., Kolonin M.G. (2018). Cancer as a Matter of Fat: The Crosstalk between Adipose Tissue and Tumors. Trends Cancer.

[83] Cuiffo B.G., Karnoub A.E. (2012). Mesenchymal stem cells in tumor development. Cell Adh. Migr..

[84] Eder T., Weber A., Neuwirt H., Grünbacher G., Ploner C., Klocker H., Sampson N., Eder I. (2016). Cancer-Associated Fibroblasts Modify the Response of Prostate Cancer Cells to Androgen and Anti-Androgens in Three-Dimensional Spheroid Culture. Int. J. Mol. Sci..

[85] Brancato V., Comunanza V., Imparato G., Corà D., Urciuolo F., Noghero A., Bussolino F., Netti P.A. (2017). Bioengineered tumoral microtissues recapitulate desmoplastic reaction of pancreatic cancer. Acta Biomater..

[86] Santo V.E., Estrada M.F., Rebelo S.P., Abreu S., Silva I., Pinto C., Veloso S.C., Serra A.T., Boghaert E., Alves P.M. (2016). Adaptable stirred-tank culture strategies for large scale production of multicellular spheroid-based tumor cell models. J. Biotechnol..

[87] Devarasetty M., Dominijanni A., Herberg S., Shelkey E., Skardal A., Soker S. (2020). Simulating the human colorectal cancer microenvironment in 3D tumor-stroma co-cultures in vitro and in vivo. Sci. Rep..

[88] Majety M., Pradel L.P., Gies M., Ries C.H. (2015). Fibroblasts Influence Survival and Therapeutic Response in a 3D Co-Culture Model. PLoS ONE.

[89] Chen Y.-C., Zhang Z., Fouladdel S., Deol Y., Ingram P.N., McDermott S.P., Azizi E., Wicha M.S., Yoon E. (2016). Single cell dual adherent-suspension co-culture micro-environment for studying tumor–stromal interactions with functionally selected cancer stem-like cells. Lab Chip.

[90] Nakamura H., Sugano M., Miyashita T., Hashimoto H., Ochiai A., Suzuki K., Tsuboi M., Ishii G. (2019). Organoid culture containing cancer cells and stromal cells reveals that podoplanin-positive cancer-associated fibroblasts enhance proliferation of lung cancer cells. Lung Cancer.

[91] Pape J., Magdeldin T., Stamati K., Nyga A., Loizidou M., Emberton M., Cheema U. (2020). Cancer-associated fibroblasts mediate cancer progression and remodel the tumouroid stroma. Br. J. Cancer.

[92] Lugo-Cintrón K.M., Gong M.M., Ayuso J.M., Tomko L.A., Beebe D.J., Virumbrales-Muñoz M., Ponik S.M. (2020). Breast Fibroblasts and ECM Components Modulate Breast Cancer Cell Migration through the Secretion of MMPs in a 3D Microfluidic Co-Culture Model. Cancers.

[93] Zhao H., Jiang E., Shang Z. (2021). 3D Co-culture of Cancer-Associated Fibroblast with Oral Cancer Organoids. J. Dent. Res..

[94] Ishii K., Nakagawa Y., Matsuda C., Katoh D., Ichishi M., Shirai T., Hirokawa Y., Fujiwara M., Sugimura Y., Watanabe M. (2021). Heterogeneous induction of an invasive phenotype in prostate cancer cells by coculturing with patient-derived fibroblasts. J. Cell. Biochem..

[95] Chen S., Giannakou A., Wyman S., Gruzas J., Golas J., Zhong W., Loreth C., Sridharan L., Yamin T.-T., Damelin M. (2018). Cancer-associated fibroblasts suppress SOX2-induced dysplasia in a lung squamous cancer coculture. Proc. Natl. Acad. Sci. USA.

[96] Hume R.D., Berry L., Reichelt S., D’Angelo M., Gomm J., Cameron R.E., Watson C.J. (2018). An Engineered Human Adipose/Collagen Model for In Vitro Breast Cancer Cell Migration Studies. Tissue Eng. Part A.

[97] Buschhaus J.M., Humphries B.A., Eckley S.S., Robison T.H., Cutter A.C., Rajendran S., Haley H.R., Bevoor A.S., Luker K.E., Luker G.D. (2020). Targeting disseminated estrogen-receptor-positive breast cancer cells in bone marrow. Oncogene.

[98] Lewis K.J.R., Hall J.K., Kiyotake E.A., Christensen T., Balasubramaniam V., Anseth K.S. (2018). Epithelial-mesenchymal crosstalk influences cellular behavior in a 3D alveolus-fibroblast model system. Biomaterials.

[99] Saini H., Rahmani Eliato K., Veldhuizen J., Zare A., Allam M., Silva C., Kratz A., Truong D., Mouneimne G., LaBaer J. (2020). The role of tumor-stroma interactions on desmoplasia and tumorigenicity within a microengineered 3D platform. Biomaterials.

[100] Norberg K.J., Liu X., Moro C.F., Strell C., Nania S., Blümel M., Balboni A., Bozóky B., Heuchel R.L., Löhr J.M. (2020). A novel pancreatic tumour and stellate cell 3D co-culture spheroid model. BMC Cancer.

[101] Lee J.-H., Kim S.-K., Khawar I.A., Jeong S.-Y., Chung S., Kuh H.-J. (2018). Microfluidic co-culture of pancreatic tumor spheroids with stellate cells as a novel 3D model for investigation of stroma-mediated cell motility and drug resistance. J. Exp. Clin. Cancer Res..

[102] Wong C.-W., Han H.-W., Tien Y.-W., Hsu S. (2019). Biomaterial substrate-derived compact cellular spheroids mimicking the behavior of pancreatic cancer and microenvironment. Biomaterials.

[103] Hwang H.J., Oh M.-S., Lee D.W., Kuh H.-J. (2019). Multiplex quantitative analysis of stroma-mediated cancer cell invasion, matrix remodeling, and drug response in a 3D co-culture model of pancreatic tumor spheroids and stellate cells. J. Exp. Clin. Cancer Res..

[104] Bersini S., Jeon J.S., Dubini G., Arrigoni C., Chung S., Charest J.L., Moretti M., Kamm R.D. (2014). A microfluidic 3D in vitro model for specificity of breast cancer metastasis to bone. Biomaterials.

[105] Wang X., Sun L., Maffini M.V., Soto A., Sonnenschein C., Kaplan D.L. (2010). A complex 3D human tissue culture system based on mammary stromal cells and silk scaffolds for modeling breast morphogenesis and function. Biomaterials.

[106] Morales D., Lombart F., Truchot A., Maire P., Hussein M., Hamitou W., Vigneron P., Galmiche A., Lok C., Vayssade M. (2019). 3D Coculture Models Underline Metastatic Melanoma Cell Sensitivity to Vemurafenib. Tissue Eng. Part A.

[107] Lamberti M.J., Morales Vasconsuelo A.B., Ferrara M.G., Rumie Vittar N.B. (2020). Recapitulation of Hypoxic Tumor–stroma Microenvironment to Study Photodynamic Therapy Implications. Photochem. Photobiol..

[108] Lamberti M.J., Rettel M., Krijgsveld J., Rivarola V.A., Vittar N.B.R. (2019). Secretome profiling of heterotypic spheroids suggests a role of fibroblasts in HIF-1 pathway modulation and colorectal cancer photodynamic resistance. Cell. Oncol..

[109] Kock A., Bergqvist F., Steinmetz J., Elfman L.H.M., Korotkova M., Johnsen J.I., Jakobsson P., Kogner P., Larsson K. (2020). Establishment of an in vitro 3D model for neuroblastoma enables preclinical investigation of combined tumor-stroma drug targeting. FASEB J..

[110] Houshmand M., Soleimani M., Atashi A., Saglio G., Abdollahi M., Zarif M.N. (2017). Mimicking the Acute Myeloid Leukemia Niche for Molecular Study and Drug Screening. Tissue Eng. Part C Methods.

[111] Dhimolea E., Simoes R.D.M., Kansara D., Weng X., Sharma S., Awate P., Liu Z., Gao D., Mitsiades N., Schwab J.H. (2021). Pleiotropic Mechanisms Drive Endocrine Resistance in the Three-Dimensional Bone Microenvironment. Cancer Res..

[112] Toh Y.-C., Lim T.C., Tai D., Xiao G., van Noort D., Yu H. (2009). A microfluidic 3D hepatocyte chip for drug toxicity testing. Lab Chip.

[113] Lee J.Y., Chaudhuri O. (2021). Modeling the tumor immune microenvironment for drug discovery using 3D culture. APL Bioeng..

[114] Marzagalli M., Ebelt N.D., Manuel E.R. (2019). Unraveling the crosstalk between melanoma and immune cells in the tumor microenvironment. Semin. Cancer Biol..

[115] Shelton S.E., Nguyen H.T., Barbie D.A., Kamm R.D. (2021). Engineering approaches for studying immune-tumor cell interactions and immunotherapy. iScience.

[116] Park N., Pandey K., Chang S.K., Kwon A.-Y., Cho Y.B., Hur J., Katwal N.B., Kim S.K., Lee S.A., Son G.W. (2020). Preclinical platform for long-term evaluation of immuno-oncology drugs using hCD34+ humanized mouse model. J. Immunother. Cancer.

[117] Yin L., Wang X.-J., Chen D.-X., Liu X.-N., Wang X.-J. (2020). Humanized mouse model: A review on preclinical applications for cancer immunotherapy. Am. J. Cancer Res..

[118] Tian H., Lyu Y., Yang Y.-G., Hu Z. (2020). Humanized Rodent Models for Cancer Research. Front. Oncol..

[119] Boucherit N., Gorvel L., Olive D. (2020). 3D Tumor Models and Their Use for the Testing of Immunotherapies. Front. Immunol..

[120] Rocha S., Basto A.P., Ijsselsteijn M.E., Teles S.P., Azevedo M.M., Gonçalves G., Gullo I., Almeida G.M., Maqueda J.J., Oliveira M.I. (2021). Immunophenotype of Gastric Tumors Unveils a Pleiotropic Role of Regulatory T Cells in Tumor Development. Cancers.

[121] Majedi F.S., Hasani-Sadrabadi M.M., Thauland T.J., Li S., Bouchard L.-S., Butte M.J. (2020). T-cell activation is modulated by the 3D mechanical microenvironment. Biomaterials.

[122] Courau T., Bonnereau J., Chicoteau J., Bottois H., Remark R., Assante Miranda L., Toubert A., Blery M., Aparicio T., Allez M. (2019). Cocultures of human colorectal tumor spheroids with immune cells reveal the therapeutic potential of MICA/B and NKG2A targeting for cancer treatment. J. Immunother. Cancer.

[123] Surendran V., Rutledge D., Colmon R., Chandrasekaran A. (2021). A novel tumor-immune microenvironment (TIME)-on-Chip mimics three dimensional neutrophil-tumor dynamics and neutrophil extracellular traps (NETs)-mediated collective tumor invasion. Biofabrication.

[124] Tang M., Xie Q., Gimple R.C., Zhong Z., Tam T., Tian J., Kidwell R.L., Wu Q., Prager B.C., Qiu Z. (2020). Three-dimensional bioprinted glioblastoma microenvironments model cellular dependencies and immune interactions. Cell Res..

[125] Marrella A., Dondero A., Aiello M., Casu B., Olive D., Regis S., Bottino C., Pende D., Meazza R., Caluori G. (2019). Cell-Laden Hydrogel as a Clinical-Relevant 3D Model for Analyzing Neuroblastoma Growth, Immunophenotype, and Susceptibility to Therapies. Front. Immunol..

[126] Appleton K.M., Elrod A.K., Lassahn K.A., Shuford S., Holmes L.M., DesRochers T.M. (2021). PD-1/PD-L1 checkpoint inhibitors in combination with olaparib display antitumor activity in ovarian cancer patient-derived three-dimensional spheroid cultures. Cancer Immunol. Immunother..

